# Parental Worries, Child Maltreatment Risk and Empowerment: How Are They Noticed in Child and Family Services?

**DOI:** 10.3390/children9020269

**Published:** 2022-02-16

**Authors:** Sari Johanna Lepistö, Noora Ellonen, Heidi Eveliina Rantanen, Maaret Kristiina Vuorenmaa, Mika Tapio Helminen, Eija Paavilainen

**Affiliations:** 1 Faculty of Social Sciences, Nursing Sciences, Tampere University, 33520 Tampere, Finland; heidi.rantanen@tuni.fi; 2 Faculty of Social Sciences, Tampere University, 33014 Tampere, Finland; noora.ellonen@tuni.fi; 3 Finnish Institute for Health and Welfare, 00271 Helsinki, Finland; maaret.vuorenmaa@thl.fi; 4 Tays Reseach Services, Tampere University Hospital, 33520 Tampere, Finland; mika.helminen@tuni.fi; 5 Faculty of Social Sciences, Health Sciences, Tampere University, 33520 Tampere, Finland; eija.paavilainen@tuni.fi; 6 South Ostrobothnia Hospital District, 60220 Seinäjoki, Finland

**Keywords:** child maltreatment, empowerment, worries, risk, child and family services

## Abstract

Parental empowerment has been related to their well-being and self-efficacy. Learning more about the signs describing child maltreatment risk are crucial for the welfare of children and families. The aim of this study was to assess the risk of child maltreatment (CM) and related worrying factors of parents and associations between the CM risk, worries and parental empowerment. The study is based on self-report surveys administered to parents in primary health care and hospital settings. The risk of CM and related worrying factors were measured by the Brief Child Abuse Potential Inventory (BCAP) from 453 parents. Family empowerment was measured by The Generic Family Empowerment Scale (G-FES). Parents expressed worries such as loneliness and distress (20%), feelings of persecution (9%), family conflict (17%), rigidity (21%) and financial insecurity (4%). The BCAP found 27 parents with increased risk. Parents with CM risk expressed more empowerment in connection to services for their child and family. It is crucial to discuss worries in child and family services before they raise the risk level. Tools such as the BCAP are useful in systematically identifying the child maltreatment risk and parental worries under discussion, offering possibilities for preventing child maltreatment and increasing well-being of children.

## 1. Introduction

The anticipation and prevention of problems in the life control of families and increasing awareness about parental worries are crucial for the well-being of children and families. Without adequate support, parental worries such as loneliness and rigidity in child rearing can lead to risk conditions of child maltreatment (CM). Therefore, services should be arranged on the basis of children’s and families’ needs [1]. The latest research, both in Finland [2,3,4,5,6,7] and globally [8], shows that children and young people experience a wide range of maltreatment at home, including physical, emotional and sexual violence, neglect and witnessing violence between parents. Preventing CM by knowing more about parental worries and possible family risk situations is a priority for multi-professional child and family services [9,10,11].

CM is defined as physical abuse, including violent punishment, sexual and emotional violence and neglect of children under 18 years of age carried out by their caregivers [12]. For example, according to Paavilainen and others [13] family functioning in CM families (meaning interaction, stability, flexibility and roles) is inferior to other families. It could also be connected with poorer family well-being. The parental risk of CM could be estimated by known risk factors in family life [14,15,16,17,18,19]. In addition, by determining a family’s level of risk, asking parents their concerns and supporting family functioning, the risk of CM can possibly be reduced and prevented [20,21]. Future research should focus more on basic child and family services to identify those who have a risk of CM and who need more support [22]. Earlier research evidence shows that the accumulation of risk factors puts children at risk for maltreatment. Children exposed to a cumulative risk at least once in early development, compared to those with no risk exposure, showed a significantly higher likelihood of an abnormal level of problem behaviors [23,24]. Therefore, it is necessary to develop ways to notice parental worries before escalating.

One of the Health 2020 priority areas is “investing in health through a life-course approach and empowering people” emphasizing comprehensive, continuous, ethical, safe and sustainable health services [25]. Parental empowerment signifies parents’ sense of confidence, for instance, dealing with services use with their children [26,27,28]. Strong empowerment is associated with parents’ resilience to demands and their confidence to make decisions and take actions positively affecting their families [29,30,31]. Empowerment is also connected with internal resources [32], creating the conditions that allow the individual to participate and make decisions about their own family, organizations and society. Empowerment varies in different life situations at the levels of individual, family, social network, service system, municipality and society [26,32,33]. In those situations, empowerment includes a parent’s sense of knowledge, understanding and rights associated with their child’s services and their sense of confidence in collaborating with professionals, participating in decision making and acting in ways that ensure access to the requisite services [26,34].

The aim of this study was to describe parental worrying factors linked to risk of CM and how parents express empowerment connected to health care services, especially for children. We aimed to increase professionals’ knowledge and provide early support to prevent CM. The objectives of the study are to describe worries, CM risk and empowerment from a parental perspective. Furthermore, associations between background variables, maltreatment worries, risk and family empowerment are described.

## 2. Materials and Methods

In this study, the short form of the Child Abuse Potential Inventory (BCAP) [14,15,35] was used to assess the risk of CM and worrying factors involved in families in the general population. The Generic Family Empowerment Scale (G-FES) was used to measure parental empowerment connected to health care services, especially for children [36,37].

Between January 2017 and March 2018, the BCAP and G-FES were delivered to parents prior to an ordinary appointment to a primary maternity health care clinic, a child health care clinic and a maternity outpatient clinic, or during the child’s admission in the general pediatric ward, the surgical ward or the neonatal intensive care unit in the hospital. Surveys were given to parents expecting children and to parents with children of different ages, and could be answered together or separately. In all contexts, after the parents returned the questionnaire, all the worrying items were discussed together with the parent, documented by the nurse and relevant support actions were commenced. Nurses, midwifes and primary health care nurses were trained beforehand on how to use BCAP, encounter and start a solution-based dialogue with the parent, document appropriately sensitive issues and contact and start supportive actions [38].

The study protocol was approved by the Research Ethics Committee of Pirkanmaa Hospital District in Southern Finland (R11198H). Permission was also obtained from the research sites. The information stressed that participation in the study was completely voluntary and free to withdraw at any time [39,40].

### 2.1. Background Variables

Background questions are listed in Table 1.

### 2.2. Brief Child Abuse Potential Inventory (BCAP)

The BCAP [35] is a short version of the CAP [14] and has been shown to be a valid screening measure, especially for child physical abuse potential on several countries [35,41], including Finland [15,16,42,43]. According to Milner and Crouch [42], the BCAP is the most psychometrically sound scale of all CAP short versions that have been developed. In the Finnish general population, internal reliability of the BCAP was good (Cronbach’s alpha 0.770) [6]. The BCAP was created to retain as much shared variance with the full measure as possible, a stable factor structure and a useful validity scale, and to maximize the BCAP’s predictive validity [35]. The BCAP is a forced-choice (agree–disagree) self-report questionnaire that consists of 25 items and 9 validity items. Seven descriptive factor scales are included: distress, family conflict, rigidity, happiness, feelings or persecution, loneliness, and financial insecurity. Validation of the BCAP among the Finnish general population resulted in a structure with five sub-scales: loneliness and distress (9 items), problems with others (4), family conflict (3), rigidity (3) and financial insecurity (2) [6]. Factors used combine measuring the risk–no risk cut-off point. In the BCAP, each item receives a score of 1 [35,41].

In our study, the BCAP was utilized in a novel way: as a basis for a solution-based dialogue using single items as discussion topics. This was carried out as part of the comprehensive family care to provide early support to prevent CM. We aimed at understanding in detail worries expressed by parents, for example, if a parent agreed with the item “other people have made my life hard”, a professional would ask “can you describe in what ways do people make your life hard?” We also counted the risk level, but we did not use this as the only basis of clinical care. We wanted to know if the novel way to use BCAP is useful or not. We also only found 27 families with CM risk in our data and did not want to make conclusions that were too detailed based on the results of 27 families.

### 2.3. The Generic Family Empowerment Scale (G-FES)

Empowerment was measured by the Generic Family Empowerment Scale (G-FES) [36,37], which is based on the Family Empowerment Scale developed for families of children with emotional disabilities [26]. This study used one dimension approach (service situation) to the generic version of the FES (12 items, α = 0.84). It refers to parents’ confidence to obtain and influence the services that their children need and use. The G-FES has a 5-point Likert-type rating scale (1 = fully disagree to 5 = fully agree). Higher scores indicate higher levels of empowerment. The original FES [26,36] and the G-FES [44] have proved to be valid and reliable instruments.

### 2.4. Analysis

In the G-FES scale, the variables were combined into three answer categories: “disagree” (1 = fully disagree and 2 = partly disagree), “no opinion” (3 = no disagree, no agree) and “agree” (4 = partly agree and 5 = fully agree). Concerning the BCAP, the risk values were categorized “no risk” (values between 0–5) and “risk” (values over 5). The long CAP Inventory cut-off point of 100 has been used in Finnish setting, which is almost 20% of the maximum score (486). Based on the same reasoning, the appropriate cut-off point for the BCAP would be five [6]. Loneliness and distress, problems with others, family conflict, rigidity and financial insecurity were categorized as zero-point “no worries” and one-point “worries” [6]. Descriptive analysis (e.g., describing of worries) was used for these categorized variables, but comparative analysis was used for continuing variables.

Descriptive statistics were used to examine the demographic data. Statistical analyses were performed using SPSS version 22.0. Frequencies and percentages were computed to describe the data. Fisher’s exact test was used to examine the associations between the central variables in relation to family risk. The level of statistical significance was set at *p* < 0.05. Association between variables was calculated with correlation analysis, Mann–Whitney U test and Kruskall–Wallis H test, depending on variables distribution.

## 3. Results

### 3.1. Description of Participants

The BCAP and the G-FES was delivered to 759 parents, of whom, 464 returned the questionnaires. From a reliability point of view, we applied the rule used in the CAP Inventory, that if a respondent has more than 10% missing responses, the questionnaire should be considered invalid. Therefore, those 11 parents having at least three missing responses were excluded from further analysis [6]. Of the final sample of the 453 parents who answered the questionnaire, 373 mothers and 80 fathers were included. The mothers were mostly between 31 and 35 years of age (31%). Most of the parents had one to three children (79% mothers and 83% fathers). Some parents were expecting their first child (See Table 1).

### 3.2. Parental Worries and Risk of Child Maltreatment

There were 426 parents with no CM risk and 27 parents with risk (value over 5) (see Table 1). Though the risk seemed to increase with age and seemed to be more common in parents expecting their first child, statistical significances were not found. The results of the BCAP showed that 20% of parents reported being worried about loneliness and distress (76% had no worries), 9% reported worries of having problems with others (90% had no worries), 17% reported family conflict (83% had no worries), 21% reported rigidity (74% had no worries) and 4% reported financial insecurity (96% had no worries).

Statistically significant (*p* < 0.001) differences were found between parents in BCAP sub-scales as follows: loneliness and distress (no risk, M = 0.258; risk, M = 3.958), problems with others (no risk, M = 0.072; risk, M = 1.708), family conflict (no risk, M = 0.154; risk, M = 1.042), rigidity (no risk, M = 0.246; risk, M = 0.750) and financial insecurity (no risk, M = 0.026; risk, M = 0.250). There was no association found with gender (see Table 2). Older parents more often experienced family conflict (less than 25 years old, M = 0.09; 26–30 years old, M = 0.12; 31–35 years old, M = 0.27; more than 36 years, old M = 0.26; *p* = 0.013). Parents without children reported having more problems with others (no child, M = 0.76; one child, M = 0.05; two or three children, M = 0.21; four or more children, M = 0.24; *p* < 0.001) and more children in a family was associated with family conflict (no child, M = 0.00; one child, M = 0.14; two or three children, M = 0.30; four or more children, M = 0.23; *p* = 0.007). In no-risk parents, there was an association between age and problems with others (less than 25 years old, M = 0.05; 26–30 years old, M = 0.01; 31–35 years old, M = 0.10; more than 36 years old, M = 0.11; *p* = 0.032) and family conflict (M = 0.10, M = 0.07, M = 0.24 and M = 0.15, respectively; *p* = 0.005). In no-risk parents, there was also association between number of children and problems with others (no child, M = 0.33; one child, M = 0.03; two or three children = 0.07; four or more children, M = 0.13; *p* = 0.003). No-risk parents without children reported having more problems with others (see Table 2).

### 3.3. Parental Empowerment

Parental empowerment was quite high in all parents (see Table 3). There was only statistical significance between no-risk and risk parents answers; “When necessary, I take the initiative in looking for services for my child and family”. Risk parents in this situation were more empowered (*p* = 0.023).

No statistically significant association was seen between empowerment and background variables in the risk parents’ group (see Table 4).

### 3.4. Associations between Family Empowerment and Risk of Child Maltreatment

There was some association between BCAP sub-scales and empowerment (see Table 5). Loneliness and distress were associated with five empowerment answers. In all cases, those who were not lonely, were more empowered. Problems with others was associated only for the answer: “my opinion is just as important as a professional’s opinion” (different opinion, M = 4.13; same opinion, M = 3.80; *p* = 0.009). Family conflict was associated with four answers, and in all cases, the same opinion for problems with family was associated with less empowerment. Rigidity was associated with two answers: “able to work with agencies and professionals” (different opinion, M = 4.74; same opinion, M = 4.64; *p* = 0.038) and “stay in regular contact with professionals” (different opinion, M = 4.26; same opinion, M = 4.06; *p* = 0.014). In both cases, rigidity was associated with less empowerment. Financial insecurity was associated with four answers (see Table 5). In these cases, insecurity was associated with less empowerment.

## 4. Discussion

According to the results of the study, parental empowerment concerning their participation in their children’s services was considered to be good. Mothers felt more empowered than fathers in service situations; however, mothers were also the majority of respondents participating the survey. This is well recognized in Finland [37], and there is a clear need to enhance fathers’ attendance in family services. Families with no child or one child were more able to make good decisions and wanted professionals to ask their opinion. Families with more children were able to work with professionals and stay in regular contact with them. Empowerment increased for families when they knew the steps required when needing help; they could make good decisions and had a good understanding of the service systems. In earlier studies, these associations have been contradictory [37,44,45,46,47,48]. According to [49], parents appreciate listening, having their views and feelings acknowledged and getting practical support.

Our data included 27 parents with CM risk. The BCAP seems to work well in finding CM risk. However, the number of families with CM risk in our data is so small that no conclusions can clearly be reached. The BCAP has not previously been used in the sub-scale item level, but in this study, variation in parental worries in item level besides just counting the risk point value was explored. This scale was used to show how parental worries have been expressed already before the risk level cut-off point. The scale was aimed to find worries that should be identified to help parents before they reach the risk level. This way of using the scale developed interesting results and showed that under the risk cut-off point, there were already worries, such as loneliness or rigidity in child rearing, that were important issues to discuss with parents. All items as parents express them are useful in starting discussion with parents. In screening parental maltreatment risk only, the BCAP total score should be considered. Van Looveren et al. [50] also considers this scale useful for assessing CM risk in a systematic manner. During the COVID-19 pandemic, some new studies have shown that families are now lonelier than evert [51,52]. Loneliness was also one of the risk conditions of CM in our study. There have also been shortcomings in receiving support from professionals in child and family services [53]. This may even decrease families’ welfare and cause polarization.

However, in this study, it was found that in searching for worries by items, the BCAP can be useful in universal services where all parents with children visit. Parents’ answers to the items advance starting discussion concerning parent welfare, e.g., based on a parent telling their feelings alone or having rigid child-rearing thoughts. In clinical practice with parents, starting a discussion based on the worries that parents express by answering the scale items is easier than starting discussion about the risk level based on a mere number that is over a cut-off point. The number itself does not tell us anything about family life; there still remains the need to ask parents to talk about the situation as the basis of further intervening and support [54]. And when the professional who starts a discussion with parent(s) about their life situation sees that that there are several family worries, perhaps over the counted risk level, there is always the possibility to procure multi-professional assessment and support. It is also necessary to start a discussion about CM risk, if the cut-off point is reached.

There are, however, some limitations to this research. The data were collected in seven different units in two different areas of Finland as a part of a research and development project. There is no complete estimate of total sample or power analysis. The response rate of families was 61%, with fathers participating well. However, due to the small number of respondents, the response rate for some variables was quite low, which made it difficult to complete statistical analyses and draw strong conclusions. It is challenging to motivate respondents to participate in this kind of study, which deals with sensitive family issues. However, it is crucial to obtain research evidence on parents’ own experiences concerning their family life and services intended to help them and their children. The number of families with CM risk could be even higher among those families who did not participate in this study. Even anonymous feedback from families concerning the services was very challenging to obtain. This can be due to sensitivity of the topic under our study.

## 5. Conclusions

Nursing and other professionals working with parents or with those who are expecting children, need tools such as the BCAP, to recognize parents’ worries and families in risk situations. In the review on evaluating risks, Gillingham [9] concluded that risk to the safety and welfare of children is difficult to predict and manage. In our present study, we showed that it is possible to identify parents with CM risk using the BCAP. This instrument can be used in many kinds of services meant for families with children. It can serve as a starting point for discussion and as part of a systematic assessment of a family’s situation to help recognize the worries of parents. In this way, it is possible to have an impact on those worries before they escalate in order to increase the well-being of children.

## Figures and Tables

**Table 1:** Demographic characteristics of respondents (N = 453).

	Mother		Father		No-Risk Family		Risk Family		*p*-Value
Variable Value	n	%	n	%	n	%	n	%	
Gender									0.445
Mother	373	82			349	94	24	6	
Father			80	18	77	96	3	4	
Age									0.891
less than 25 years	40	11	7	9	45	96	2	4	
26–30 years old	106	29	20	25	119	94	7	6	
31–35 years old	114	31	22	28	128	94	8	6	
more than 36 years old	109	20	31	39	130	93	10	7	
Number of children									0.121
none at all	13	4	4	6	15	88	2	12	
one	118	34	29	40	142	97	5	3	
two or three	153	45	31	43	167	91	17	9	
four or more	59	17	8	11	64	96	3	4	

*p*-value calculated by Fisher’s exact test, risk families comparison with no-risk families.

**Table 2:** BCAP sum variables’ association with background variables, *p*-value (N = 453).

	All Answers			No-Risk Families			Risk Families		
Sum Variables	Gender ^a^	Age ^b^	Number of Children ^b^	Gender ^a^	Age ^b^	Number of Children ^b^	Gender ^a^	Age ^b^	Number of Children ^b^
Loneliness and distress	0.277	0.645	0.828	0.470	0.264	0.798	0.352	0.838	0.191
Problems with others	0.781	0.051	0.000 **	0.185	0.032 *	0.003 **	0.705	0.902	0.073
Family conflict	0.138	0.013 *	0.007 **	0.193	0.005 **	0.091	0.635	0.187	0.190
Rigidity	0.463	0.216	0.205	0.451	0.111	0.198	0.395	0.940	0.386
Financial insecurity	0.918	0.187	0.521	0.882	0.108	0.296	0.743	0.690	0.454

^a^ Mann–Whitney U test, ^b^ Kruskall–Wallis H test. * *p* < 0.05, ** *p* < 0.01.

**Table 3:** Parental empowerment (N = 453).

	All Answers		All Answers			No-Risk Families			Risk Families			*p*-Value
	Mean	Sd	1–2 %	3 %	4–5 %	1–2 %	3 %	4–5 %	1–2 %	3 %	4–5 %	
About your child’s services *	4.28	0.47										0.880
I feel that I have a right to approve all services my child receives.	4.62	0.69	2.2	3.5	93.4	2.3	3.5	93.2	0.0	3.7	96.3	0.291
I know the steps to take when I am concerned my child is receiving poor services.	4.17	0.85	5.7	9.5	84.5	5.6	9.9	84.3	7.4	3.7	88.9	0.731
I make sure that professionals understand my opinions about what services my child needs.	4.40	0.74	1.8	9.5	87.9	1.9	9.6	87.6	0.0	7.4	92.6	0.151
I am able to make good decisions about what services my child needs.	4.52	0.72	2.2	4.0	92.9	2.1	4.0	93.0	3.7	3.7	92.6	0.455
I am able to work with agencies and professionals to decide what services my child needs.	4.71	0.60	0.9	3.8	94.7	0.7	3.8	94.8	3.7	3.7	92.6	0.970
I make sure I stay in regular contact with professionals who are providing services to my child.	4.23	0.88	2.9	19.6	75.3	3.1	20.2	74.4	0.0	11.1	88.9	0.733
My opinion is just as important as a professional’s opinion in deciding what services my child needs.	4.10	0.98	11.5	6.8	81.0	11.5	6.8	81.0	11.1	7.4	81.5	0.193
I tell professionals what I think about services being provided to my child.	3.99	0.91	5.7	20.1	73.1	5.9	20.2	72.8	3.7	18.5	77.8	0.963
I know what services my child needs.	4.25	0.74	2.4	9.3	87.2	2.3	9.6	86.9	3.7	3.7	92.6	0.391
When necessary, I take the initiative in looking for services for my child and family.	4.63	0.65	1.5	2.9	95.1	1.6	2.8	95.1	0.0	3.7	96.3	0.023 *
I have a good understanding of the service system that my child is involved in.	3.96	0.94	10.8	11.9	76.6	10.1	11.5	77.7	22.2	18.5	59.3	0.186
Professionals should ask me what services I want for my child.	3.82	0.92	6.2	30.5	61.8	6.3	31.0	61.0	3.7	22.2	74.1	0.192

1–2: fully or partly disagree; 3: no disagree, no agree; 4–5: fully or partly agree. * G-FES scale based on Vuorenmaa [36,37]. *p*-value calculated by Mann–Whitney U test, risk families comparison with no risk.

**Table 4:** Family empowerment association between background variables (N = 453).

	All Answers			No-risk Families			Risk Families		
Sum Variables	Gender ^a^	Age ^b^	Number of Children ^b^	Gender ^a^	Age ^b^	Number of Children ^b^	Gender ^a^	Age ^b^	Number of Children ^b^
Empowerment scale *	0.022 *	0.568	0.747	0.020 *	0.568	0.854	0.969	0.857	0.548
I feel that I have a right to approve all services my child receives.	0.284	0.389	0.379	0.237	0.455	0.296	0.392	0.314	0.502
I know the steps to take when I am concerned my child is receiving poor services.	0.853	0.843	0.818	0.844	0.693	0.652	1.000	0.355	0.762
I make sure that professionals understand my opinions about what services my child needs.	0.019 *	0.467	0.034 *	0.018 *	0.276	0.066	0.926	0.087	0.551
I am able to make good decisions about what services my child needs.	0.190	0.472	0.011 *	0.139	0.474	0.012 *	0.631	0.992	0.496
I am able to work with agencies and professionals to decide what services my child needs.	0.168	0.139	0.220	0.183	0.174	0.208	0.710	0.348	0.625
I make sure I stay in regular contact with professionals who are providing services to my child.	<0.001 **	0.002 **	0.013 *	<0.001 **	0.001 **	0.008 **	0.548	0.296	0.235
My opinion is just as important as a professional’s opinion in deciding what services my child needs.	0.484	0.034*	0.175	0.374	0.077	0.100	0.493	0.287	0.501
I tell professionals what I think about services being provided to my child.	0.077	0.321	0.556	0.076	0.315	0.461	0.901	0.990	0.983
I know what services my child needs.	0.057	0.610	0.151	0.065	0.436	0.185	0.485	0.731	0.695
When necessary, I take the initiative in looking for services for my child and family.	0.114	0.690	0.207	0.058	0.598	0.357	0.484	0.362	0.270
I have a good understanding of the service system that my child is involved in.	0.709	0.844	0.388	0.871	0.750	0.228	0.263	0.726	0.391
Professionals should ask me what services I want for my child.	0.384	0.270	0.039 *	0.332	0.430	0.040 *	0.486	0.467	0.245

^a^ Mann–Whitney U test, ^b^ Kruskall–Wallis H test. * G-FES scale based on Vuorenmaa [36,37]. * *p* < 0.05, ** *p* < 0.01.

**Table 5:** Family empowerment association between BCAP variables (N = 453).

Sum Variables	Loneliness and Distress	Problems with Others	Family Conflict	Rigidity	Financial Insecurity
	Correlation *p*-Value	Correlation *p*-Value	Correlation *p*-Value	Correlation *p*-Value	Correlation *p*-Value
Empowerment scale “	−0.147 **	−0.006	−0.112 *	0.007	−0.089
I feel that I have a right to approve all services my child receives.	0.016	0.030	−0.015	0.063	0.031
I know the steps to take when I am concerned my child is receiving poor services.	−0.160 **	0.010	−0.137 **	−0.009	−0.055
I make sure that professionals understand my opinions about what services my child needs.	−0.053	0.044	−0.052	0.041	−0.011
I am able to make good decisions about what services my child needs.	−0.117 *	0.016	−0.176 **	0.018	0.023
I am able to work with agencies and professionals to decide what services my child needs.	−.087	0.046	−0.090	−0.100 *	0.023
I make sure I stay in regular contact with professionals who are providing services to my child.	−0.074	0.029	0.002	−0.120 *	−0.058
My opinion is just as important as a professional’s opinion in deciding what services my child needs.	−0.108 *	−0.124 **	−0.043	0.045	−0.103 *
I tell professionals what I think about services being provided to my child.	−0.076	−0.029	−0.051	0.010	−0.031
I know what services my child needs.	−0.079	−0.042	−0.093 *	0.028	−0.059
When necessary, I take the initiative in looking for services for my child and family.	−0.115 *	−0.034	−0.053	−0.084	−0.100 *
I have a good understanding of the service system that my child is involved in.	−0.148 **	−0.030	−0.101 *	0.037	−0.107 *
Professionals should ask me what services I want for my child.	−0.042	0.068	0.014	0.020	−0.101 *

Spearman correlation: ** Correlation is significant at the 0.01 level (2-tailed), * Correlation is significant at the 0.05 level (2-tailed). “ G-FES scale based on Vuorenmaa [36,37].

## Data Availability

Sharing the data compromises ethical standards as it is sensitive data from families. The data included worries and risks, which the parents, besides other private issues on family life, offered to researchers in their answers. They were assured their answers would not be shown to any other parties, except for the research team. Accordingly, the authors are not able to share the data.

## References

[1] Haanpää L., Kuula M., Hakovirta M. (2019). Social relationships, child poverty and children’s life satisfaction. Soc. Sci..

[2] Lepistö S. (2010). Nuorten Kokema Perheväkivalta. Malli Hyvinvoinnista ja Selviytymisestä (Adolescents Experiences of Domestic Violence. Model of Well-Being and Coping). Ph.D. Thesis.

[3] Ellonen N., Kääriäinen J., Sariola H., Helweg-Larsen K., Larsen H. (2011). Adolescents’ experiences of parental violence in Danish and Finnish families: Comparative perspective. J. Scand. Stud. Crime Crime Prev..

[4] Lepistö S., Paavilainen E. (2013). The association between child maltreatment and coping strategies among Finnish 9th graders. Child Welfare.

[5] Peltonen K., Ellonen N., Pösö T., Lucas S. (2014). Mothers’ self-report violence toward their children: A multifaceted risk analysis. Child Abuse Negl..

[6] Ellonen N., Rantanen H., Lepistö S., Helminen M., Paavilainen E. (2019). The use of the Brief Child Abuse Potential Inventory on the normal population in Finland. Scand. J. Prim. Health Care.

[7] Leppäkoski T., Vuorenmaa M., Paavilainen E. (2021). Psychological and physical abuse towards four-year-old children as reported by their parents: A national Finnish survey. Child Abus. Negl..

[8] Gilbert R., Kemp A., Thoburn J., Sidebotham P., Radford L., Glaser D., MacMillan H. (2009). Recognizing and responding to child maltreatment. Lancet.

[9] Gillingham P. (2006). Risk assessment in child protection: Problem rather than solution?. Aust. Soc. Work.

[10] Vuorenmaa M., Halme N., Kaunonen M., Åstedt-Kurki P., Perälä M.-L. (2017). Determinants of maternal and paternal empowerment: Exploring the role of childhood adversities. Eur. J. Public Health.

[11] Langevin R., Marshall C., Kingsland  E. (2021). Intergenerational cycles of maltreatment: A scoping review of psychosocial risk and protective factors. Trauma Violence Abus..

[12] INSPIRE Seven Strategies for Ending Violence against Children. Word Health Organization: Geneve, Switzerland, 2016. http://apps.who.int/iris/bitstream/10665/207717/1/9789241565356-eng.pdf.

[13] Paavilainen E., Åstedt-Kurki P., Paunonen M., Laippala P. (2001). Risk factors of child maltreatment within the family: Towards a knowledgeable base of family nursing. Nurs. Stud..

[14] Milner J. (1986). The Child Abuse Potential Inventory Manual.

[15] Milner J. (1994). Assessing physical child abuse risk: The Child Abuse Potential Inventory. Clin. Psychol. Rev..

[16] Milner J.S., Crouch J.L. (2012). Psychometric characteristic of translated versions of the Child Abuse Potential Inventory. Psychol. Viol..

[17] Ayers S., Bond R., Webb R., Miller P., Bateson K. (2019). Perinatal mental health and risk of child maltreatment: A systematic review and meta-analysis. Child Abus. Negl..

[18] Assink M., Spruit A., Schuts M., Lindauer R., van der Put C.E., Stams G.J.M. (2018). The intergenerational transmission of child maltreatment: A three level meta-analysis. Child Abus. Negl..

[19] Mulder T.M., Kuiper K.C., van der Put C.E., Stams G.J.M., Assink M. (2018). Risk factors for child neglect: A meta-analytic review. Child Abus. Negl..

[20] Barlow J., Davis H., McIntosh E., Jarrett P., Mockford C., Stewart-Brown S. (2007). Role of home visiting in improving parenting in families at risk of abuse and neglect: Results of a multicentre randomised controlled trial and economic evaluation. Arch. Dis. Child.

[21] Paavilainen E., Flinck A. (2015). Efficient Methods for Identifying Child Maltreatment in Social and Health Care. A Clinical Guideline. http://www.hotus.fi/system/files/SUOSITUS_lasten_kaltoinkohtelu_ENGLANTI.pdf.

[22] Lepistö S., Ellonen N., Helminen M., Paavilainen E. (2016). The family health, functioning, social support, and child maltreatment risk of families expecting a baby. J. Clin. Nurs..

[23] Patwardhan I., Duppong Hurley K., Thompson R.W., Mason W.A., Ringle J.L. (2017). Child maltreatment as a function of cumulative family risk: Findings from the intensive family preservation program. Child Abuse Negl..

[24] Wallander J., Berry S., Atattoa-Carr P., Peterson E.R. (2019). Patterns of exposure to cumulative risk through age 2 and associations with problem behaviors at age 4.5: Evidence from growing up in New Zealand. J. Abnorm. Child Psychol..

[25] WHO (2015). European Strategic Directions for Strengthening Nursing and Midwifery towards 2020 Goals.

[26] Koren P.E., DeChillo N., Friesen B.J. (1992). Measuring empowerment in families whose members have emotional disabilities: A brief questionnaire. Rehabil. Psychol..

[27] Warren Z., McPheeters M.L., Sathe N., Foss-Feig J.H., Glasser A., Veenstra-VanderWeele J. (2011). A systemic review of early intensive intervention for autism spectrum disorders. Pediatrics.

[28] Kerppola J. (2021). Parental Empowerment in Child and Family Services.

[29] Liu C.-H., Chao Y.-H., Huang C.-M., Wei F.-C., Chien L.-Y. (2010). Effectiveness of applying empowerment strategies when establishing a support group for parents of preterm infants. J. Clin. Nurs..

[30] Lambard A.M., Jurkowski J.M., Lawson H.A., Davison K.K. (2013). Family ecological predictors of physical activity parenting in low-income families. Behav. Med..

[31] Martins T.U., Zerbetto S.R., Dupas G. (2013). Empowerment mechanisms used by the family of a child with cleft li and palate to a resilient path. Cien Cuis Saude.

[32] Raivio H., Karjalainen J., Era T. (2013). Osallisuus ei ole keino tai väline, palvelut ovat (Involvement is not a means or tool, services are). Osallisuus—Oikeutta vai Pakkoa? (Involvement—A Right or an Obligation?).

[33] Mendez J.L. (2010). How can parents get involved in preschool? Barriers and engagement in education by ethnic minority parents of children attending Head Start. Cultur. Divers. Ethnic Minor. Psychol..

[34] Vuorenmaa M., Perälä M.-L., Halme N., Kaunonen M., Åstedt-Kurki P. (2015). Associations between family characteristics and parental empowerment in the family, family service situations and the family service system. Child Care Health Dev..

[35] Ondersma S., Caffin M., Mullins S., LeBreton J. (2005). A brief form of the Child Abuse Potential Inventory: Development and Validation. J. Clin. Child Adolesc. Psychol..

[36] Vuorenmaa M., Halme N., Åstedt-Kurki P., Kaunonen M., Perälä M.-L. (2014). The validity and reliability of the Finnish Family Empowerment Scale (FES): A survey of parents with small children. Child Care Health Dev..

[37] Vuorenmaa M. (2016). Äitien ja Isien Osallisuus Perheessä ja Lasten Palveluissa sekä Osallisuuteen Yhteydessä Olevat Tekijät (Maternal and Paternal Empowerment in the Family and Services That the Child Uses and Factors That Are Related to Parental Empowerment). Ph.D. Thesis.

[38] Leppäkoski T., Rantanen H., Helminen M., Paavilainen E. (2019). How Training Impact the Identification and Discussion of the Risk of Child Maltreatment: A Finnish Follow-Up Study. Glob. J. Health Sci..

[39] Holloway I., Wheeler S. (2002). Qualitative Research in Nursing.

[40] Paavilainen E., Lepistö S., Flinck A. (2014). Ethical issues in family violence research in healthcare settings. Nurs. Ethics.

[41] Walker C., Davies J. (2012). A cross-cultural validation of the Brief Child Abuse Potential Inventory (BCAP). J. Fam. Viol..

[42] Milner J.S., Crouch J.L., Campbell J.C., Messing J.T. (2017). Child Physical Abuse Risk Assessment: Parent and family evaluations. Assessing Dangerousness—Domestic Violence Offenders and Child Abusers.

[43] Ellonen N., Lepistö S., Helminen M., Paavilainen E. (2017). Cross-cultural validation of the Child abuse potential inventory in Finland: Preliminary findings of the study among parents expecting a baby. J. Soc. Serv. Res..

[44] Wakimizu R., Fujioka H., Yoneyama A., Iejima A., Miyamoto S. (2011). Factors associated with the empowerment of Japanesefamilies raising a child with developmental disorders. Res. Dev. Disab..

[45] Resendez M., Quist R., Matshazi D. (2000). A longitudinal analysis of family empowerment and client outcomes. J. Child Fam. Stud..

[46] Hintermair M. (2006). Parental resources, parental stress, and socioemotional development of deaf and hard of hearing children. J. Deaf Stud. Deaf Educ..

[47] Castillo J., Welch G., Sarver C. (2011). Fathering: The relationship between fathers’ residence, fathers’ sociodemographic characteristics, and father involvement. Matern. Child Health J..

[48] Melnyk B.M., Oswalt K.L., Sidora-Arcoleo K. (2014). Validation and psychometric properties of the Neonatal Intensive Care Unit Parental Beliefs Scale. Nurs. Res..

[49] Bekaert S., Paavilainen E., Schecke H., Baldaccino A., Jouet E., Zabłocka-Żytka L., Bachi B., Bartoli F., Carrà G., Cioni R.M. (2021). Family members’ perspectives on child protective services, a metasynthesis of the literature. Child Youth Serv. Rev..

[50] Van Looveren N., Glazemakers I., van Grootel L., Fransen E., van West D. (2017). Assessment of physical child abuse risk in parents with children referred to child and adolescent psychiatry. Child Abuse Rev..

[51] Klemetti R., Vuorenmaa M., Ervasti E., Helakorpi S., Lammi-Taskula J. (2021). Vauvaperheiden vanhempien kokemat huolet ja muutokset sosiaalisissa suhteissa ja jaksamisessa koronaepidemian aikana. [Concerns and changes in social relationships and coping experienced by parents of baby families during the corona epidemic]. Sos. Aikakauslehti Soc. Med. J..

[52] Kovacs B., Caplan N., Grob S., King M. (2021). Social networks and loneliness during the COVID-19 pandemic. Socius.

[53] Lammi-Taskula J., Vuorenmaa M., Aunola K., Sorkkila M. Matalan kynnyksen sosiaalipalvelut lapsiperheiden tukena ja palveluiden käyttöCOVID-19-epidemian aikana. [Low-Threshold Social Services in Support of Families with Children and Use of Services during the COVID-19 Epidemic]. Terveyden ja hyvinvoinnin laitos. Tutkimuksesta tiiviisti, 15/2020. http://urn.fi/URN:ISBN:978-952-343-522-3.

[54] Flinck A., Rantanen H., Paavilainen E. (2019). Äitien kokemuksia neuvolapalveluista ja neuvolan kyselylomakkeista. Analyysi äitien kirjoituksista sosiaalisessa mediassa. [Mothers’ experiences with counseling services and counseling questionnaires. Analysis of mothers’ writings on social media]. Yhteisk. Soc. Politic.

